# Acetamiprid Affects Destruxins Production but Its Accumulation in *Metarhizium* sp. Spores Increases Infection Ability of Fungi

**DOI:** 10.3390/toxins12090587

**Published:** 2020-09-11

**Authors:** Monika Nowak, Przemysław Bernat, Julia Mrozińska, Sylwia Różalska

**Affiliations:** 1 Department of Industrial Microbiology and Biotechnology, Faculty of Biology and Environmental Protection, University of Łódź, 90–237 Łódź, Poland; monika.nowak@unilodz.eu (M.N.); przemyslaw.bernat@biol.uni.lodz.pl (P.B.); 2 Scientific Students Group “SKN Bio-Mik”, Faculty of Biology and Environmental Protection, University of Łódź, 90–237 Łódź, Poland; julia.mrozia@gmail.com

**Keywords:** entomopathogens, mycoinsecticides, secondary metabolites, insect pathogenesis, acetamiprid accumulation

## Abstract

*Metarhizium* sp. are entomopathogenic fungi that inhabit the soil environment. Together, they act as natural pest control factors. In the natural environment, they come into contact with various anthropogenic pollutants, and sometimes, they are used together and interchangeably with chemical insecticides (e.g., neonicotinoids) for pest control. In most cases, the compatibility of entomopathogens with insecticides has been determined; however, the influence of these compounds on the metabolism of entomopathogenic fungi has not yet been studied. Secondary metabolites are very important factors that influence the fitness of the producers, playing important roles in the ability of these pathogens to successfully parasitize insects. In this study, for the first time, we focus on whether the insecticide present in the fungal growth environment affects secondary metabolism in fungi. The research revealed that acetamiprid at concentrations from 5 to 50 mg L^−1^ did not inhibit the growth of all tested *Metarhizium* sp.; however, it reduced the level of 19 produced destruxins in direct proportion to the dosage used. Furthermore, it was shown that acetamiprid accumulates not only in plant or animal tissues, but also in fungal cells. Despite the negative impact of acetamiprid on secondary metabolism, it was proofed to accumulate in *Metarhizium* spores, which appeared to have a stronger infectious potential against mealworm *Tenebrio molitor*, in comparison to the insecticide or the biological agent alone.

## 1. Introduction

Synthetic insecticides are important pesticides for both agricultural and domestic pest control. Among them, neonicotinoids (e.g., acetamiprid, imidacloprid, thiacloprid) are the most extensively used worldwide because of their high effectiveness in controlling crop and domestic pests [1]. They account for more than 25% of the global insecticide market and are now being considered as a replacement for many existing conventional insecticide classes [2]. Neonicotinoids’ popularity is largely due to their physicochemical properties, high effectiveness, low resistance, and the fact that they are less harmful to mammals compared to other insecticides. For example, to protect fruit plants against pests, up to 3 g per 100 m^2^ of a popular pest control product (e.g., Mospilan 20 SP) is used with acetamiprid as the active substance are used and, as a consequence, acetamiprid penetrates the soil and bioaccumulates. According to data from the European Food Safety Authority (EFSA), ^14^C-acetamiprid was identified as the major constituent of the radioactive residues in all plant parts (the study included eggplants, apples, carrots and cabbage) in an amount of 30–90% within 14–90 days after the last application [3]. The ability of neonicotinoids to accumulate in plants is known to increase the probability of environmental contamination and exposure to nontarget organisms [3,4,5]. In addition, despite the lack of recognition of acetamiprid as a compound persisting in soil, its degradation in environmental conditions has been found to last up to 43 days [3].

Among all methods of insect control, biological methods deserve special attention. *Metarhizium* sp., the common insect pathogens in wildlife, are very efficient bioinsecticides usually applied in practice [6]. Their effectiveness against insects depends mainly on their infective potential, i.e., the ability to produce extracellular lytic enzymes and secondary metabolites [7,8]. While the extracellular enzymes are well studied, equally important secondary metabolites, which play critical roles in the ability of *Metarhizium* to successfully parasitize their hosts and ultimately contribute to the success or failure of these fungi as biological control agents, are quite often neglected [9]. Entomopathogenic fungi produce a variety of bioactive metabolites including >40 cyclic hexadepsipeptides destruxins (dtxs) [8]. It is suggested that in attacked insects, dtxs induce paralysis and muscle contraction via muscle depolarization by the direct opening of Ca^2+^ channels in the membrane [10,11]. Besides their insecticidal activity, dtxs have also potential as pharmaceuticals showing antiviral, antitumor, cytotoxic, immunosuppressant or antiproliferative effects [12,13]. However, due to the endophytic properties of *Metarhizium* sp., the presence of dtxs can be found in, e.g., potatoes [14], maize and strawberries [15], causing dtxs to enter the food chain and pose a threat to human health.

The aim of this work was to determine the influence of acetamiprid on the growth and the secondary metabolism of *Metarhizium,* which is considered as a significant factor during the pest infection process. Furthermore, we checked whether acetamiprid could be accumulated by *Metarhizium*, how it affected the production of dtxs, and whether it affected the ability of fungi to infect insects. This study could help to understand the potential risks of a harmful influence of acetamiprid on soil-inhabiting fungi and their infectious potential, which plays an important role in maintaining the ecological balance.

## 2. Results

### 2.1. Fungal Biomass Yield of Metarhizium sp. in the Presence of Acetamiprid

**Figure 1:**
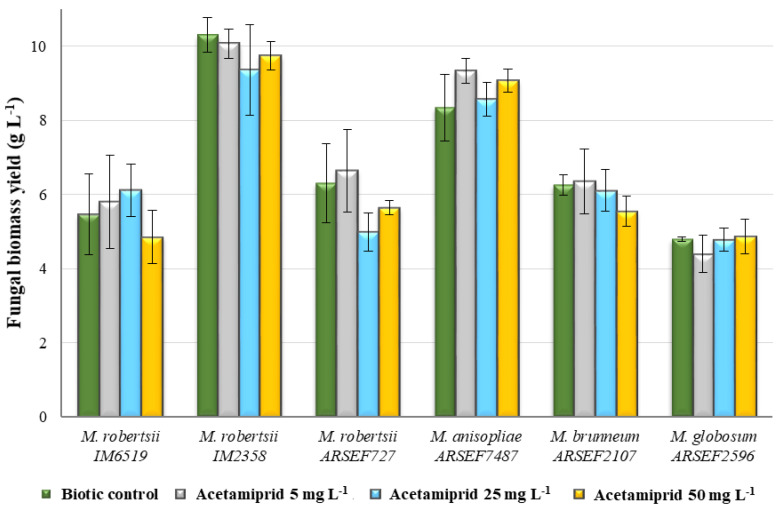
Influence of acetamiprid at concentrations of 5, 25 and 50 mg L^−1^ on fungal biomass yield of *Metarhizium* sp. One-way ANOVA was used for investigations of statistical significance. All differences are statistically insignificant (*p* > 0.05).

Among the tested strains, *M. anisopliae* and *M. robertsii* IM2358 were the best growing species, while *M. brunneum* and *M. robertsii* ARSEF727 grew at a similar medium rate (biomass yield about 6 g L^−1^). The slowest growing species was *M. globosum*. It turned out that acetamiprid did not inhibit growth in any of the tested strains, even at the highest concentration of 50 mg L^−1^ (*p* > 0.05) (Figure 1). Literature data also provides information that acetamiprid has no harmful effect on conidia germination and production or vegetative growth in *M. anisopliae*—strain E9 (ESALQ/USP) [16]. The lack of toxic effect was also confirmed in the presented study. 

### 2.2. Quantitative Analyses of the Content of Acetamiprid in Metarhizium Fungal Cultures

In this study, the fungal ability to eliminate acetamiprid added to cultures at concentrations of 5, 25 and 50 mg L^−1^ was verified (Figure S1). None of the tested *Metarhizium* sp. showed the highly effective ability to remove acetamiprid from the Czapek Dox culture medium after seven days of incubation. At the concentration of 5 mg L^−1^, a slight elimination capacity was demonstrated for *M. brunneum* and *M. robertsii* IM6519 (the average removal rate reached 29 and 24%, respectively). In a situation where five times more acetamiprid was added to the fungal culture, a loss was observed in the samples of all tested strains. *M. anisopliae*, *M. globosum* and *M. brunneum* removed respectively 31, 25 and 24% of acetamiprid from the culture medium. In the case of the other species, the substrate elimination was below 20%. In the fungal cultures, where the insecticide concentration was 50 mg L^−1^ there was no loss of more than 20% of the insecticide for any species. However, studies of the content of acetamiprid separately in the mycelium and culture medium showed that it was accumulated in the fungal cells. 

**Figure 2:**
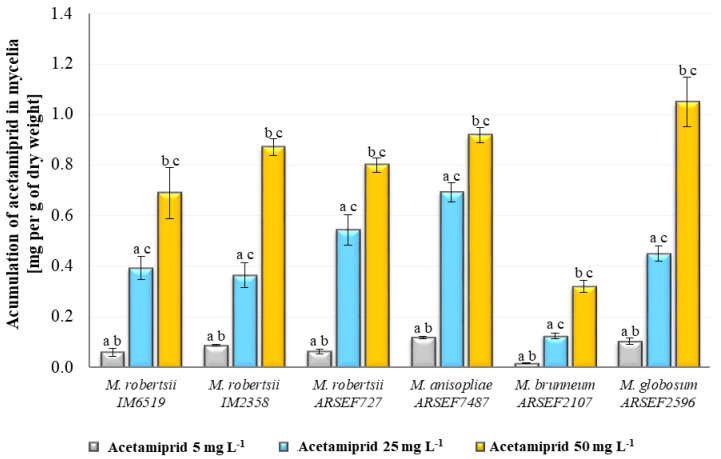
Accumulation of acetamiprid at concentrations of 5, 25 and 50 mg L^−1^ in the mycelium of *Metarhizium* sp. One-way ANOVA and Tukey’s test were used for investigations of statistical significance. (**a**–**c**) *p* < 0.05. Statistically significant differences between samples at individual concentrations within the species. (**a**) Between samples with acetamiprid at concentrations of 5 and 25 mg L^−1^; (**b**) between samples with acetamiprid at concentrations of 5 and 50 mg L^−1^; (**c**) between samples with acetamiprid at concentrations of 25 and 50 mg L^−1^.

This insecticide has proved the ability to accumulate, inter alia, in the tissues of plants where, through translocation, it can even move from the roots to the shoots [17,18]. It was found that due to its good solubility in water, acetamiprid has a strong toxic effect on aquatic organisms where it bioaccumulates by sorption mechanisms characteristic for compounds with high polarity [19,20]. Although acetamiprid accumulation ability has been described for several different species, in this paper, we present for the first time that entomopathogens, which are often used interchangeably or alternatively with various insecticides in agriculture, can also accumulate this compound, without metabolizing it inside the cells (during seven days of incubation). Herein, the amount of acetamiprid on the mycelium dry weight (mg g^−1^) was directly proportional to the increasing concentration (*p* < 0.05) (Figure 2). This trend was similar for each tested species while *M. brunneum* exhibited the highest accumulation potential. The rest of the strains showed statistically significant differences in the amounts of acetamiprid accumulated in the cells at the three concentrations used. At the concentration of 25 mg L^−1^ in *M. anisopliae* the highest amount of the accumulated insecticide (0.69 mg per g of dry weight) was observed (*p* < 0.05), but it is worth noting that this species had been previously found to have high biomass yield (Figure 1)*. M. robertsii* IM6519, *M. robertsii* IM2358 and *M. globosum* accumulated in the cells’ comparable amounts of acetamiprid (approximately 0.36–0.45 mg per g of dry weight, *p* > 0.05). By comparison, 0.12 mg per g of dry weight was found in *M. brunneum* (*p* < 0.05). The situation changed for the acetamiprid concentration of 50 mg L^−1^ in the fungal culture and the amount of the toxic substrate accumulated in the mycelium leveled out (Figure 2). The largest quantity was detected for *M. globosum* (1.05 mg per g of dry weight), which was different from the other species (except *M. anisopliae*, *p* > 0.05). In all *M. robertsii* strains and the *M. anisopliae* strain studied, the acetamiprid amounts determined per g of dry weight were comparable.

### 2.3. Analyses of Destruxins in Fungal Cultures of Metarhizium sp.

According to literature data, *M. anisopliae*, *M. robertsii* and *M. brunneum* species have genes responsible for the production of dtxs [8]. Dtxs in *M. globosum* were also marked in this work, but their content was very low compared to the other species (in the order of 0.002 and 0.004 mg L^−1^ for dtx A and dtx B, respectively), so this species was excluded from further analyses. *M. robertsii* ARSEF727 had the lowest content of dtxs in the biotic control compared to the other species. The expression of genes responsible for the production of specific units making up the dtxs structure might not be as high as in the case of the other strains [8]. It cannot be said that the low concentrations of dtxs in *M. robertsii* ARSEF727 and *M. globosum* were caused by the poor growth of these fungi, because their growth rate was similar to that of *M. brunneum* and *M. robertsii* IM6519, which turned out to be the species with the highest content of dtxs in the biotic controls. Interestingly, *M. anisopliae*, in which the synthesis of dtxs has been accurately described, did not turn out to be the best producer [21]. *M. robertsii* IM2358 was also found to have higher levels of dtxs than *M. anisopliae*.

According to literature data, none of the fungal species has the capacity to produce all 39 types of dtxs, but *M. anisopliae* produces the majority of them [8]. In all tested strains, except *M. globosum*, 19 dtxs were determined. Due to the lack of chromatography standards, accurate quantitative analyses were performed only for dtx A and B. Therefore, the amounts of dtx A and B and the other types were described separately. It is worth noting that dtx A and dtx B are the main metabolites and occur in higher concentrations compared to other dtxs [22], which was also confirmed in this work.

**Figure 3:**
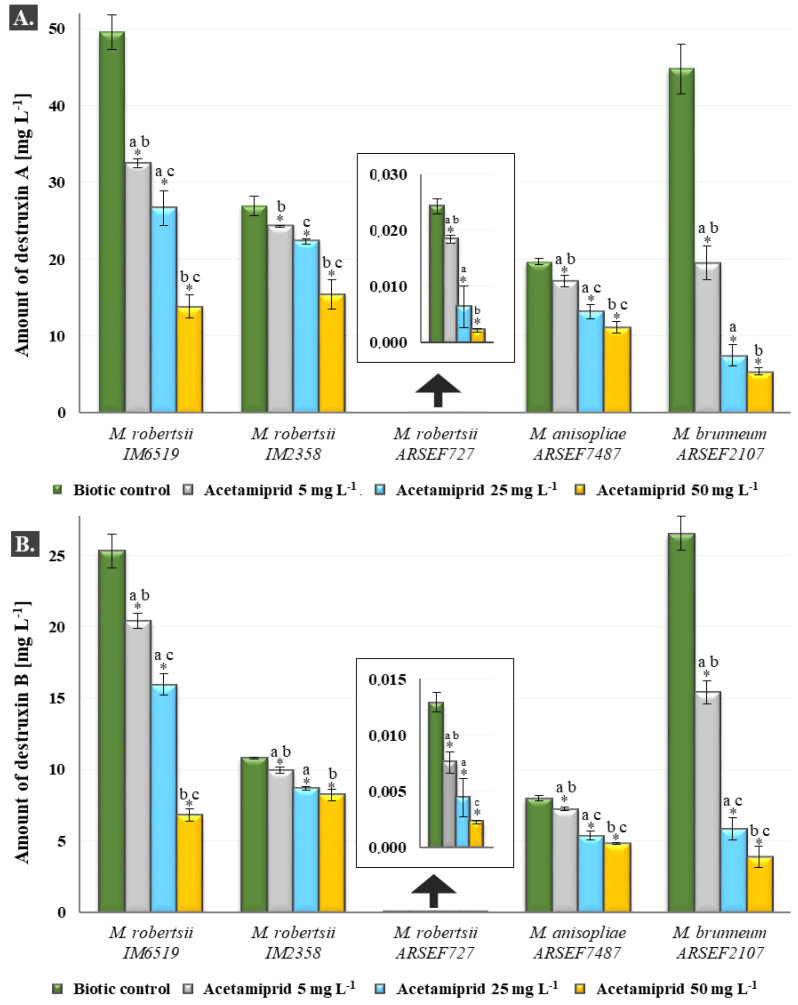
Effect of acetamiprid at concentrations of 5, 25 and 50 mg L^−1^ on the amounts of destruxins A (**A**) and B (**B**) produced by *Metarhizium* species. One-way ANOVA and Tukey’s test were used for investigations of statistical significance. * *p* < 0.05. Statistically significant differences between samples with acetamiprid at concentrations of 5, 25 and 50 mg L^−1^ and their biotic controls within the species; (a–c) *p* < 0.05—statistically significant differences between samples at individual concentrations within the species. (a) Between samples with acetamiprid at concentrations of 5 and 25 mg L^−1^; (b) between samples with acetamiprid at concentrations of 5 and 50 mg L^−1^; (c) between samples with acetamiprid at concentrations of 25 and 50 mg L^−1^.

It was checked whether, despite the lack of the influence of acetamiprid on the growth of the tested fungi, this insecticide affected the secondary metabolism. As mentioned above, three concentrations of acetamiprid (5, 25 and 50 mg L^−1^) were examined. It turned out that the lowest concentration caused disturbances in the synthesis of the secondary metabolites of *Metarhizium* (*p* < 0.05). The use of higher doses of acetamiprid contributed to a gradual reduction in the amount of detected dtxs. Declines in the content of dtxs A and B were quite proportional (Figure 3). For *M. brunneum* there were no statistically significant differences between the amounts of dtx A at concentrations of 25 and 50 mg L^−1^. The differences between all the concentrations used, which were determined for dtx B were statistically significant. The amounts of dtx A for *M. robertsii* IM2358 did not differ significantly between the concentrations of 5 and 25 mg L^−1^, and for dtx B between the concentrations of 25 and 50 mg L^−1^. No statistically significant differences between the concentrations of 25 and 50 mg L^−1^ for dtx A and B were found for *M. robertsii* ARSEF727.

The most harmful effect of acetamiprid on the production of dtxs was noted for *M. brunneum,* despite the fact that the growth rate and amounts of dtxs in the biotic sample were similar to those observed for *M. robertsii* IM6519. At the concentration of 5 mg L^−1^, the contents of dtxs A and B were 56.43 and 41.93% lower than in the biotic controls, respectively. The highest dose of the insecticide resulted in a very large reduction in the contents of dtxs A and B to 11.99 and 14.58% of the biotic control, respectively. As mentioned above, *M. brunneum* accumulated in the mycelium the lowest quantities of acetamiprid per g of dry weight (Figure 2). It seems that *M. brunneum* defended itself against the presence of the insecticide in the mycelium. This could have been the reason why acetamiprid so heavily influenced the contents of dtxs in this strain. Dtxs production in *M. robertsii* ARSEF727 and *M. robertsii* IM6519 was also inhibited at the highest concentration of the insecticide and for dtx A the amounts were 90.99 and 72.09% lower than in the biotic control, and for dtx B 82.53 and 73.15%, respectively (Figure 3).

Comparable decreases in the contents of dtxs A and B were observed for *M. anisopliae* and *M. robertsii* IM2358 strains. With the increase in the acetamiprid concentration, the amounts of dtx A were lower by 10.04, 17.33 and 43.05% for *M. robertsii* IM2358 and by 13.10, 33.24 and 43.61% for *M. anisopliae*. It was noticed that the dose of 25 mg L^−1^ was more toxic to *M. anisopliae* than to *M. robertsii* IM2358. The concentration of dtx B for *M. anisopliae* decreased in a similar way to the content of dtx A (9.49, 33.05 and 40.03% less than in the biotic control, with the increase in the acetamiprid concentration). For *M. robertsii* IM2358 at the of concentrations 5 and 25 mg L^−1^ the decreases were similar to dtx A (7.94 and 19.68% less than in the biotic control, respectively), while at the concentration of 50 mg L^−1^, the content of dtx B decreased by 24.21% (the reduction was almost two-fold smaller than for dtx A).

**Figure 4:**
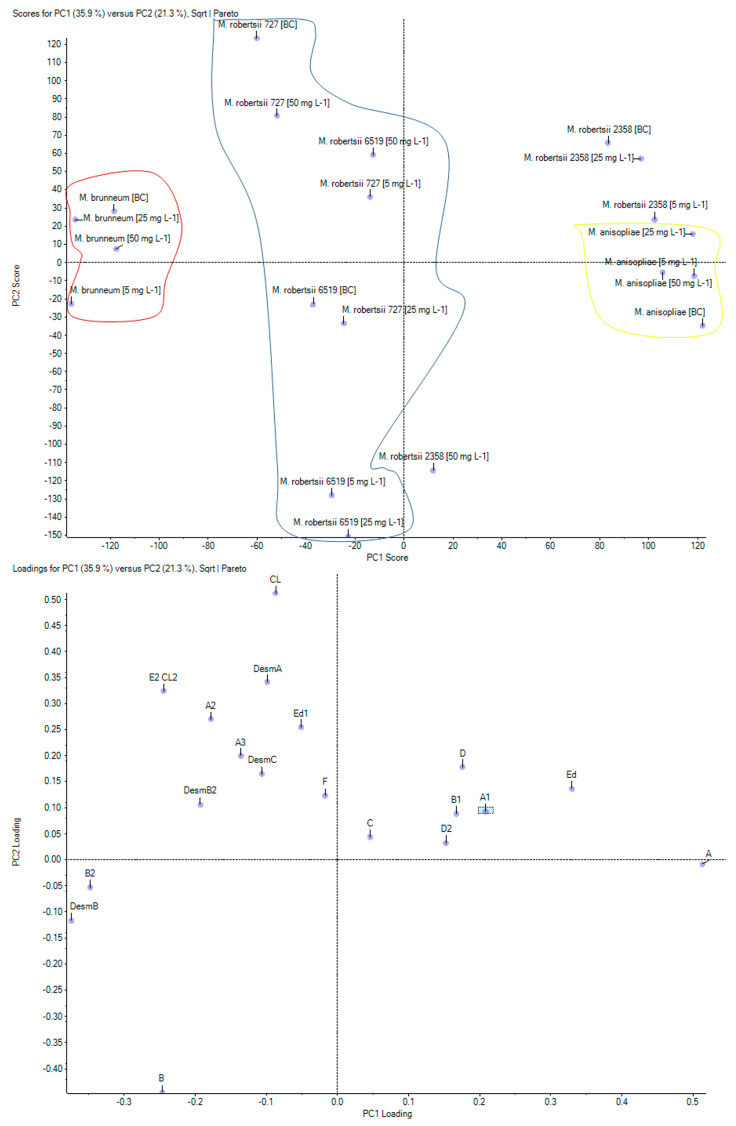
Results of principal component analysis (PCA) on the profile of destruxins of *Metarhizium* species in samples without the addition of acetamiprid and with acetamiprid at concentrations of 5, 25 and 50 mg L^−1^. PC1 against PC2 scores chart (**top**); PC1 against PC2 loadings chart (**bottom**).

The levels of the other 17 dtxs, for which chromatographic standards are not available, were estimated based on the chromatographic peak areas. It turned out that the tested species differed in terms of the profile of dtxs, which was confirmed by the PCA (Figure 4). To the best of our knowledge, this kind of analysis had never been done before. 

A similar dtxs profile was obtained for *M. anisopliae* and *M. robertsii* IM2358 (Figure 4). These strains differed from the others due to the high levels of dtx B1 and dtx Ed. A superior decrease in the levels of these dtxs and dtx D was observed for *M. robertsii* IM2358 with the acetamiprid concentration of 50 mg L^−1^, hence in the PCA chart, this tested sample was distinguished and shown to migrate towards *M. robertsii* IM6519 (tested samples with acetamiprid at concentrations of 5 and 25 mg L^−1^), in which the levels of these dtxs were also low (Figure 4). The level of dtx DesmA distinguished *M. anisopliae* from *M. robertsii* IM2358, as lower values were obtained for *M. anisopliae*. The level of synthesized dtx A1 was definitely a factor differentiating *M. anisopliae* from the other tested species, because for this strain, the highest level of dtx A1 was determined. Such close proximity of *M. anisopliae* and *M. robertsii* IM2358 on the PCA chart was in line with the presented previously. These species were characterized by a similar growth (Figure 1) and the effect of acetamiprid at concentrations of 5, 25 and 50 mg L^−1^ on the decrease in the amounts of dtxs A and B (Figure 3).

The above-mentioned results of the accumulation of acetamiprid in the mycelium showed that *M. brunneum* differed from the other species because of the lowest quantity of the insecticide bound in the fungal cells (Figure 2). It turned out that the dtxs profile of this strain also contributed to its differentiation, particularly to the high levels of dtxs DesmB, B2, DesmB2 and E2CL2 and the relatively low levels of dtxs Ed and D2.

Analysis of the PCA chart was more complicated for the strains *M. robertsii* ARSEF727 and *M. robertsii* IM6519. Their profiles were quite similar; however, it was possible to see differences in the contents of individual dtxs (Figure 4). The differentiating factor for *M. robertsii* ARSEF727 was the high level of dtx Ed1, particularly for the sample with acetamiprid at the concentration of 50 mg L^−1^, where the addition of acetamiprid did not reduce the level of dtx Ed1 more than in the sample with the addition of acetamiprid at the concentration of 25 mg L^−1^. The high level of dtx E2CL2 slightly differentiated from the biotic sample with *M. robertsii* ARSEF727 from the other samples of this species. Dtx CL was at a high level in *M. robertsii* IM6519, but in the sample with acetamiprid at a concentration of 50 mg L^−1^, it definitely decreased. The close proximity of *M. robertsii* IM6519 (the biotic control and the sample with acetamiprid at a concentration of 50 mg L^−1^) to *M. robertsii* ARSEF727 was due to the high level of dtx A3. However, the low level of this dtx in the tested sample with acetamiprid at a concentration of 25 mg L^−1^ in *M. robertsii* ARSEF727 made this strain more similar to *M. robertsii* IM6519 (samples with acetamiprid at concentrations of 5 and 25 mg L^−1^).

### 2.4. Permeability of the Cell Membrane and the Content of Acetamiprid in Spores and Subcellular Fractions of the M. brunneum

*M. brunneum* was chosen to conduct an experiment on the effect of acetamiprid on the permeability of biological membranes, due to the greatest inhibition of dtxs production by this compound, even to 88% at the concentration of 50 mg L^−1^ (Figure 3). Dtxs are extracellular metabolites; however, their synthesis takes place in fungal cells [23]. The properties of the cell membrane, including permeability, potential and fluidity, have an influence on the cell secretion process [24]. Acetamiprid did not reduce the permeability of biological membranes at any of the used concentrations (*p* > 0.05). The reduced amount of dtxs determined in the samples with the addition of the toxic insecticide was not associated with a disorder of the membrane permeability system (Table S1).

Due to the high inhibition of dtxs production, it was checked whether acetamiprid accumulated in the spores of *M. brunneum*. Additionally, its amount in the cell wall and other subcellular structures of the fungus was determined. It turned out that acetamiprid accumulated in the spores (0.280 ± 0.05 µg per 10^6^ of spores), while in cell fractions almost 6-fold more acetamiprid was detected in the cell wall (27.61 ± 3.75 µg per g of dry weight) than in the other subcellular structures (4.78 ± 0.65 µg per g of dry weight).

### 2.5. Influence of Acetamiprid, Spores of M. brunneum ARSEF2107 and the Combination of Spores and Acetamiprid on the Mortality of Tenebrio molitor (Mealworm)

**Figure 5:**
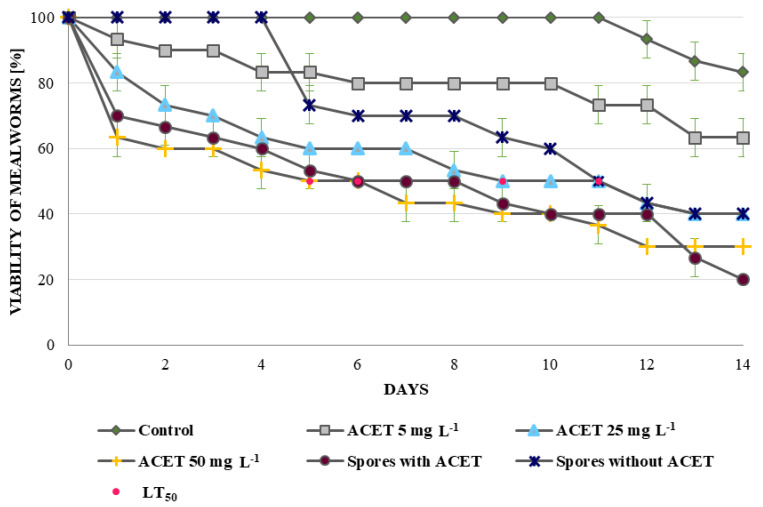
Viability of *Tenebrio molitor* mealworms treated with acetamiprid (ACET) at the concentrations of 5, 25 and 50 mg L^−1^, with *Metarhizium brunneum* spores and a combination of spores and ACET. Statistical significance was assessed by standard deviations.

Acetamiprid is used worldwide as an effective insect control agent. The dose of acetamiprid at used in these studies (5, 25 and 50 mg L^−1^) caused mortality of *T. molitor* (Figure 5).

Additionally, the action of the neonicotinoid was compared to the killing properties of *M. brunneum* spores. The obtained results of acetamiprid accumulation in the fungal spores prompted us to determine what effect could be generated by the combination of *M. brunneum* spores with the accumulated acetamiprid and whether such a mixture could be an alternative to using the chemical insecticide alone. The results were surprising because a dose of 25 µg acetamiprid (50 mg L^−1^) caused similar mealworm mortality resulting from a combination of spores with the insecticide accumulated in the amount of 140 ng, i.e., almost 180-fold less. The difference in LT_50_ was one day, and for the highest dose of acetamiprid this value was determined on the fifth day of testing, and for the combination of spores and insecticide on day six. Acetamiprid at the highest dose acted slightly faster, while at the end of the experiment it turned out that the highest mortality was achieved for the combined action of spores and acetamiprid. Similarly to chemical insecticides, entomopathogens do not kill insects immediately and their lethal effect is delayed even up to 14 days [13]. When a combination of spores and acetamiprid was used, an effect similar to that achieved by the insecticide applied alone was observed. This suggests that when using a combination of spores and acetamiprid, the mechanism of action of the insecticide is can be followed, but due to the accumulation of acetamiprid in the spores, the form of application of the toxic compound has been changed, and therefore, it could be used in a smaller amount than in a situation when it is applied as the only insect-killing agent. 

In the natural environment, acetamiprid can impair dtxs production, thereby affecting the functioning of *Metarhizium* sp. in their habitat and the fight for an ecological niche. Due to the increased infectivity of *Metarhizium* spores applied with acetamiprid, it is possible that not only pests, but also beneficial arthropod species may be exposed to this toxic action.

## 3. Conclusions

The study reveals that, although acetamiprid does not inhibit the growth of *Metarhizium* sp., it affects the metabolism of the fungus by decreasing its ability to produce dtxs. This phenomenon may have environmental implications because dtxs are produced not only during infection but can also be important for the survival of *Metarhizium* in soil. It has been proved for the first time that acetamiprid accumulates in fungi, not only in plant and animal tissues, which could have some ecological implications as well. However, in the case of *Metarhizium*, the most important finding is the fact that acetamiprid increases its infectivity as in the experiments the spores with the accumulated insecticide were shown to cause the highest mortality of the tested larvae. On the other hand, in the light of the results obtained, it cannot be conclusively stated that a decrease in the production of dtxs caused by acetamiprid does not disturb the infection process because acetamiprid is a strong insecticide whose combined action with *M. brunneum* was revealed to be the most effective in the mortality tests of *T. molitor*.

## 4. Materials and Methods

### 4.1. Chemicals and Reagents

All reagents and solvents were of analytical or liquid chromatography-mass spectrometry (LC–MS) grade and were purchased from Sigma-Aldrich (Steinheim, Germany) unless otherwise stated. Destruxin A (dtx A) from *M. anisopliae* and destruxin B (dtx B; Cayman Chemical, Ann Arbor, MI, USA) were used as chromatography standards. LC–MS grade water (Merck, Darmstadt, Germany) was used for chromatography. Fungi were cultivated on Czapek Dox broth (BD-Difco, Le Pont-de-Claix, France). Acetamiprid (99% purity) was added to the fungal cultures and was also used as a chromatography standard.

### 4.2. Microorganisms and Cultivation Conditions

Six fungal strains of the genus *Metarhizium* (*M. robertsii* IM6519, *M. robertsii* IM2358, *M. robertsii* ARSEF727, *M. anisopliae* ARSEF7487, *M. brunneum* ARSEF2107, *M. globosum* ARSEF2596) from the strains collection of the ARSEF (The Agricultural Research Service Collection of Entomopathogenic Fungal Cultures) and the Department of Industrial Microbiology and Biotechnology, University of Lodz (Poland), were used during the investigations. All strains were maintained on ZT agar slants(glucose (4 g L^−1^); Difco yeast extract (4 g L^−1^); agar (25 g L^−1^); and malt extract (6 °Blg), up to 1 L; pH 7.0) as described previously [25,26,27]. Cultures for tests with *Metarhizium* spore suspension adjusted to 1 × 10^6^ spores mL^−1^ were prepared on Czapek Dox liquid medium (total volume 40 mL in 100 mL Erlenmeyer flasks; BD-Difco, Le Pont-de-Claix, France). Acetamiprid stock dissolved in acetonitrile at the concentration of 50 mg mL^−1^ was added to fungal cultures to a final concentration of 5, 25 and 50 mg L^−1^. Additionally, abiotic controls (without fungal biomass) and biotic controls (without acetamiprid) were prepared. All samples were incubated on a rotary shaker (120 rpm) at 28 °C for 7 days. 

### 4.3. Destruxins Extraction by a Modified QuEChERS Method

After the incubation, the fungal cultures were filtrated through Whatman filter paper Number 1 (Sigma-Aldrich, Steinheim, Germany) to separate the culture media from the mycelia. The mycelia (for biomass estimation) were harvested and dried at 105 °C until a constant weight was obtained. The culture media were centrifuged for 5 min at 10,000 rpm, and subsequently, 20 mL was transferred to each 50 mL Falcon tube with 10 mL acetonitrile. After vigorous vortexing for 1 min, 3000 rpm, *QuEChERS* salts (4 g MgSO_4_; 1 g NaCl; 1 g C_6_H_5_ Na_3_O_7_·2H_2_O; 0.5 g C_6_H_6_Na_2_O_7_·1.5 H_2_O) were added and the tubes were again vortexed for 1 min. Afterwards, the tubes were centrifuged for 5 min at 8000 rpm. Eight milliliters of the upper layer was transferred to each 15 mL Falcon tube and evaporated to dryness under reduced pressure at 40 °C. After evaporation, the samples were dissolved in 5 mL LC–MS grade water (Merck, Darmstadt, Germany) and 4 mL of the extracts were cleaned on the Solid Phase Extraction Column with octadecyl sorbent C18. Subsequently, 5 mL of acetonitrile was added to rinse the metabolites bound on the sorbent, and next, 4 mL of the extract was transferred to each 15 mL Falcon tube and again evaporated to dryness as described above. After evaporation, the samples were dissolved in 2.5 mL of LC–MS grade water and 1 mL was subjected to LC–MS/MS.

### 4.4. Acetamiprid Extraction by a Modified QuEChERS Method 

The mycelia were separated from Czapek Dox medium by filtration through Whatman filter paper Number 1 (Sigma-Aldrich, Steinheim, Germany). Then, 20 mL of deionized water and 10 mL of acetonitrile were added to each mycelium sample and ultrasonic extraction was done (2 min, Am 36%, pulse for 10 s). Then, 10 mL of acetonitrile was added to the culture medium in the volume 20 mL, and ultrasonic extraction was performed as mentioned above. The subsequent procedure was the same for both types of samples. The samples were transferred to each 50 mL Falcon tube and extracted with a Ball Mill (Retch MM400, Idar-Oberstein, Germany) for 5 min and at 25/s frequency. After homogenization, *QuEChERS salts* were added and the tubes were vortexed 3000 rpm for 1 min. Subsequently, the extracts were centrifuged for 5 min at 5000 rpm. Then, 1 mL of the top layer was collected for the LC–MS/MS analysis.

### 4.5. Acetamiprid Extraction from M. brunneum Spores and Subcellular Fractions

To determine the acetamiprid concentration in spores, 7-day-old fungal cultures were filtered through the nylon net. The filtrates were centrifuged for 10 min at 10,000 rpm, and after supernatant removal, 20 mL of deionized water was added to the spores’ pellet. The number of spores were counted in the Thoma cell counting chamber. Then, 10 mL of acetonitrile was added and the *QuEChERS* procedure was done as described above.

For acetamiprid presence in cell fractions, fungal cultures were centrifuged (10 min at 10,000 rpm), and after supernatant removal, the precipitate was washed twice with 20 mL of deionized water [28]. Then, the mycelium was suspended in 20 mL of deionized water and ultrasonic extraction was performed (2 min, Am 36%, pulse for 10 s). Mycelia disintegration was controlled by cell oscopic observations. After mycelia disruption, the samples were centrifuged for 10 min at 1200 rpm. The supernatant was separated from the precipitate with the cell wall fraction, then it was transferred into Eppendorf tubes and centrifuged for 20 min at 20,000 rpm at 4 °C. Obtained precipitates were suspended in 20 mL of deionized water, then 10 mL of acetonitrile was added and the *QuEChERS* extraction procedure was performed as described above. The cell wall fraction precipitate was washed twice in 20 mL of deionized water by centrifugation for 10 min at 1200 rpm. Then, it was suspended in 20 mL of deionized water, 10 mL of acetonitrile was added and the extraction procedure was followed as described above.

### 4.6. LC–MS/MS Quantitative and Qualitative Analyses of Destruxins

Quantitative and qualitative analyses of dtxs were carried out by using LC–MS/MS (LC Agilent 1200 coupled with a tandem mass spectrometer, AB Sciex QTRAP 4500, Framingham, MA, USA). The separation was performed with a Kinetex C18 column maintained at 40 °C. Water with 5 mM ammonium formate (AF, Solvent A) and methanol with 5 mM ammonium formate (Wolvent B) were used as mobile phases at a flow rate of 0.5 mL min^−1^. The injection volume was 5 µL. The eluent gradient was conducted as follows: hold 90% A from 0 to 0.25 min, linear increase from 90% A to 90% B to 2 min, hold 90% B from 2 to 4 min, reverse to the initial conditions from 4 to 4.1 min, and maintained for column equilibration to 6.0 min.

The detection of dtxs was conducted using MS/MS with an electrospray ion source (ESI) in the positive ionization scheduled multiple reaction monitoring (sMRM) scan mode. The MRM detection window was set to 25 s. The optimized ESI parameters were as follows: CUR: 25; IS: 5000 V; TEMP: 500 °C; GS1: 50; GS2:50. MRM parameters of 19 dtxs are presented in Table S2 [29]. Dtxs were detected in the culture medium of each of the tested strains. The quantitative analyses of dtx A and dtx B were carried out using standard curves in the linearity range 2.5–100 ng mL^−1^ (*r* = 0.9997 and *r* = 0.9999, respectively). The levels of the other dtxs were determined based on chromatographic peak areas and compared between samples using principal component analysis (PCA).

### 4.7. LC–MS/MS Quantitative Analyses of Acetamiprid

Quantitative analyses of acetamiprid were carried out using LC–MS/MS (LC Agilent 1200 coupled with a tandem mass spectrometer, AB Sciex QTRAP 3200). The separation was performed with a Kinetex C18 column maintained at 40 °C. Water with 5 mM AF (solvent A) and acetonitrile with 5 mM AF and 0.1% formic acid (FA, Solvent B) were used as mobile phases at a flow rate of 0.5 mL min^−1^. The injection volume was 10 µL. The eluent gradient was conducted as follows: hold 90% A from 0 to 0.25 min, linear increase from 90% A to 90% B to 0.5 min, hold 90% B from 0.5 to 4 min, reverse to initial conditions from 4 to 4.1 min, and maintained for column equilibration to 6.0 min.

The detection of acetamiprid was conducted using MS/MS with ESI in the positive ionization MRM scan mode (223.2/126.1 *m/z*; 223.2/73.0 *m/z*). The optimized ESI parameters were as follows: CUR: 25; IS: 5500 V; TEMP: 500 °C; GS1: 50; GS2:60. The quantitation curve of acetamiprid was accomplished in the quadratic regression in the range 25–1000 ng mL^−1^ and *r* = 0.9999.

### 4.8. Permeability of the Cell Membranes

The intention was to check whether acetamiprid simply interferes with the transport of dtxs from the fungal cell to the culture medium, or its action is connected with another mechanism. The procedure was performed according to the method described by Siewiera et al., 2015 [30] with some modifications. Briefly, 1 mL each of the control samples and the tested samples with acetamiprid concentrations of 5, 25 and 50 mg L^−1^ was transferred into Eppendorf tubes and then centrifuged for 10 min at 12,000 rpm. The supernatant was removed and 1 mL of Phosphate Buffered Saline (PBS) and 2 µL of propidium iodide (stock solution 0.1 mg mL^−1^) were added to the precipitate and the mixture was vortexed for 30 s at 3000 rpm. After incubation in the dark for 5 min, the supernatant was removed. The mycelium was washed twice in PBS by centrifugation in the conditions described previously. Finally, the samples were suspended in 1 mL of PBS and propidium iodide fluorescence was measured at λ_ex_ = 540 and λ_em_ = 630 (FLUOstar Omega, BMG LABTECH, Ortenberg, Germany). The final results were presented as fluorescence intensity per mg of dry weight. 

### 4.9. Mortality Test of Larvae of Tenebrio molitor (Mealworm)

Three concentrations of acetamiprid (5, 25 and 50 mg L^−1^) and *Metarhizium* sp. spores (1 × 10^6^ spores mL^−1^) with and without accumulated acetamiprid were tested. Control samples without any stressful factors were also done. The effects of the individual variants were checked using 10 mealworms, kept in the dark in plastic boxes with holes in the lid, and the bottom was lined with tissue paper. Before starting the experiment, the larvae were fed with oat flakes, which eliminates the death of starvation. Insect vitality was assessed daily for 14 days.

### 4.10. Data Analysis

Fungal biomass estimation and quantitative analyses of acetamiprid were conducted in four repetitions, analyses of dtxs in six repetitions. Measurements of propidium iodide fluorescence were carried out in four repetitions. The mortality test of larvae of *T. molitor* was conducted in triplicates. Variabilities of samples were given as standard deviations (±SD). The one-way analysis of variance (ANOVA) and the posthoc Tukey test were used for investigations of statistical significance, using the concentrations of acetamiprid as a factor on transformed data. Scores at *p* < 0.05 were classified as significant. Statistica 13.1 (StatSoft, Tulsa, OK, USA) was used to analyze the data. The qualitative data of dtxs were submitted to PCA in orthogonal rotation and normalization using total area sums and Pareto scaling (Marker View Software 1.2.1., AB Sciex, Framingham, MA, USA).

## 5. Patents

Patent application No. P.434091 in the Patent Office of the Republic of Poland.

## Supplementary Materials

The following are available online at https://www.mdpi.com/2072-6651/12/9/587/s1, Figure S1: Elimination of acetamiprid at the concentrations of 5 (A), 25 (B) and 50 mg L^−1^ (C) after 7 days of incubation by *Metarhizium* sp. considering the residual content in the culture medium and mycelium; Table S1: Effect of acetamiprid on the permeability of the *Metarhizium brunneum* ARSEF2107 fungal cell wall; Table S2: Multiple Reaction Monitoring (MRM) parameters for determining 19 types of destruxins.

## References

[1] Han W., Tian Y., Shen X. (2018). Human exposure to neonicotinoid insecticides and the evaluation of their potential toxicity: An overview. Chemosphere.

[2] Bass C., Denholm I., Williamson M.S., Nauen R. (2015). The global status of insect resistance to neonicotinoid insecticides. Pestic. Biochem. Physiol..

[3] EFSA (European Food Safety Authority) (2016). Conclusion on the peer review of the pesticide risk assessment of the active substance acetamiprid. EFSA J..

[4] Xiong J., Wang Z., Ma X., Li H., You J. (2019). Occurrence and risk of neonicotinoid insecticides in surface water in a rapidly developing region: Application of polar organic chemical integrative samplers. Sci. Total Environ..

[5] Ihara M. (2018). Neonicotinoids: Molecular mechanisms of action, insights into resistance and impact on pollinators. Curr. Opin. Insect Sci..

[6] Lovett B., St. Leger R.J. (2018). Genetically engineering better fungal biopesticides. Pest Manag. Sci..

[7] Mondal S., Baksi S., Koris A., Vatai G. (2016). Journey of enzymes in entomopathogenic fungi. Pac. Sci. Rev. A Nat. Sci. Eng..

[8] Wang B., Kang Q., Lu Y., Bai L., Wang C. (2012). Unveiling the biosynthetic puzzle of destruxins in Metarhizium species. Proc. Natl. Acad. Sci. USA.

[9] Donzelli B.G.G., Krasnoff S.B. (2016). Molecular Genetics of Secondary Chemistry in Metarhizium Fungi. Adv. Genet..

[10] Pedras M.S.C., Irina Zaharia L.I., Ward D.E. (2002). The destruxins: Synthesis, biosynthesis, biotransformation, and biological activity. Phytochemistry.

[11] Wang X., Gong X., Li P., Lai D., Zhou L., Wang X., Gong X., Li P., Lai D., Zhou L. (2018). Structural diversity and biological activities of cyclic depsipeptides from fungi. Molecules.

[12] Liu B.L., Tzeng Y.M. (2012). Development and applications of destruxins: A review. Biotechnol. Adv..

[13] Litwin A., Nowak M., Różalska S. (2020). Entomopathogenic fungi: Unconventional applications. Rev. Environ. Sci. Biotechnol..

[14] Ríos-Moreno A., Garrido-Jurado I., Resquín-Romero G., Arroyo-Manzanares N., Arce L., Quesada-Moraga E. (2016). Destruxin A production by *Metarhizium brunneum* strains during transient endophytic colonisation of *Solanum tuberosum*. Biocontrol Sci. Technol..

[15] Taibon J., Sturm S., Seger C., Strasser H., Stuppner H. (2015). Quantitative assessment of destruxins from strawberry and maize in the lower parts per billion range: Combination of a QuEChERS-based extraction protocol with a fast and selective UHPLC-QTOF-MS assay. J. Agric. Food Chem..

[16] Neves P.M.O.J., Hirose E., Tchujo P.T., Moino J.R.A. (2001). Compatibility of entomopathogenic fungi with neonicotinoid insecticides. Neotrop. Entomol..

[17] Li Y., Long L., Yan H., Ge J., Cheng J., Ren L., Yu X. (2018). Comparison of uptake, translocation and accumulation of several neonicotinoids in komatsuna (*Brassica rapa* var. perviridis) from contaminated soils. Chemosphere.

[18] De Laet C., Matringe T., Petit E., Grison C. (2019). Eichhornia crassipes: A Powerful Bio-indicator for Water Pollution by Emerging Pollutants. Sci. Rep..

[19] Barbieri M.V., Postigo C., Guillem-Argiles N., Monllor-Alcaraz L.S., Simionato J.I., Stella E., Barceló D., López de Alda M. (2019). Analysis of 52 pesticides in fresh fish muscle by QuEChERS extraction followed by LC-MS/MS determination. Sci. Total Environ..

[20] Bartlett A.J., Hedges A.M., Intini K.D., Brown L.R., Maisonneuve F.J., Robinson S.A., Gillis P.L., de Solla S.R. (2019). Acute and chronic toxicity of neonicotinoid and butenolide insecticides to the freshwater amphipod, Hyalella azteca. Ecotoxicol. Environ. Saf..

[21] Dong T., Zhang B., Weng Q., Hu Q. (2016). The production relationship of destruxins and blastospores of Metarhizium anisopliae with virulence against Plutella xylostella. J. Integr. Agric..

[22] Ríos-Moreno A., Carpio A., Garrido-Jurado I., Arroyo-Manzanares N., Lozano-Tovar M.D., Arce L., Gámiz-Gracia L., García-Campaña A.M., Quesada-Moraga E. (2016). Production of destruxins by *Metarhizium* strains under different stress conditions and their detection by using UHPLC-MS/MS. Biocontrol Sci. Technol..

[23] Ravindran K., Akutse S., Sivaramakrishnan S., Wang L. (2016). Determination and characterization of destruxin production in Metarhizium anisopliae Tk6 and formulations for Aedes aegypti mosquitoes control at the field level. Toxicon.

[24] Bernat P., Nykiel-Szymańska J., Stolarek P., Słaba M., Szewczyk R., Ró S. (2018). 2,4-dichlorophenoxyacetic acid-induced oxidative stress: Metabolome and membrane modifications in Umbelopsis isabellina, a herbicide degrader. PLoS ONE.

[25] Różalska S., Pawłowska J., Wrzosek M., Tkaczuk C., Długoński J. (2013). Utilization of 4-n-nonylphenol by Metarhizium sp. isolates. Acta Biochim. Pol..

[26] Rózalska S., Glińska S., Długoński J. (2014). Metarhizium robertsii morphological flexibility during nonylphenol removal. Int. Biodeterior. Biodegrad..

[27] Nowak M., Soboń A., Litwin A., Różalska S. (2019). 4-n-nonylphenol degradation by the genus Metarhizium with cytochrome P450 involvement. Chemosphere.

[28] Słaba M., Szewczyk R., Bernat P., Długoński J. (2009). Simultaneous toxic action of zinc and alachlor resulted in enhancement of zinc uptake by the filamentous fungus Paecilomyces marquandii. Sci. Total Environ..

[29] Arroyo-Manzanares N., Diana Di Mavungu J., Garrido-Jurado I., Arce L., Vanhaecke L., Quesada-Moraga E., De Saeger S. (2017). Analytical strategy for determination of known and unknown destruxins using hybrid quadrupole-Orbitrap high-resolution mass spectrometry. Anal. Bioanal. Chem..

[30] Siewiera P., Bernat P., Rózalska S., Długoński J. (2015). Estradiol improves tributyltin degradation by the filamentous fungus Metarhizium robertsii. Int. Biodeterior. Biodegrad..

