# Supplementary material for: Acetamiprid Affects Destruxins Production but Its Accumulation in Metarhizium sp. Spores Increases Infection Ability of Fungi
Source: Toxins (Basel). 2020 Sep 11;12(9):587. doi: 10.3390/toxins12090587 (PMC7551442; doi:10.3390/toxins12090587)
Supplement: Supplementary file 1 [file toxins-12-00587-s001.pdf]

Supplementary Materials: Acetamiprid Affects Destruxins Production but Its Accumulation in *Metarhizium* sp. Spores Increases Infection Ability of Fungi

Monika Nowak, Przemysław Bernat, Julia Mrozińska and Sylwia Różalska

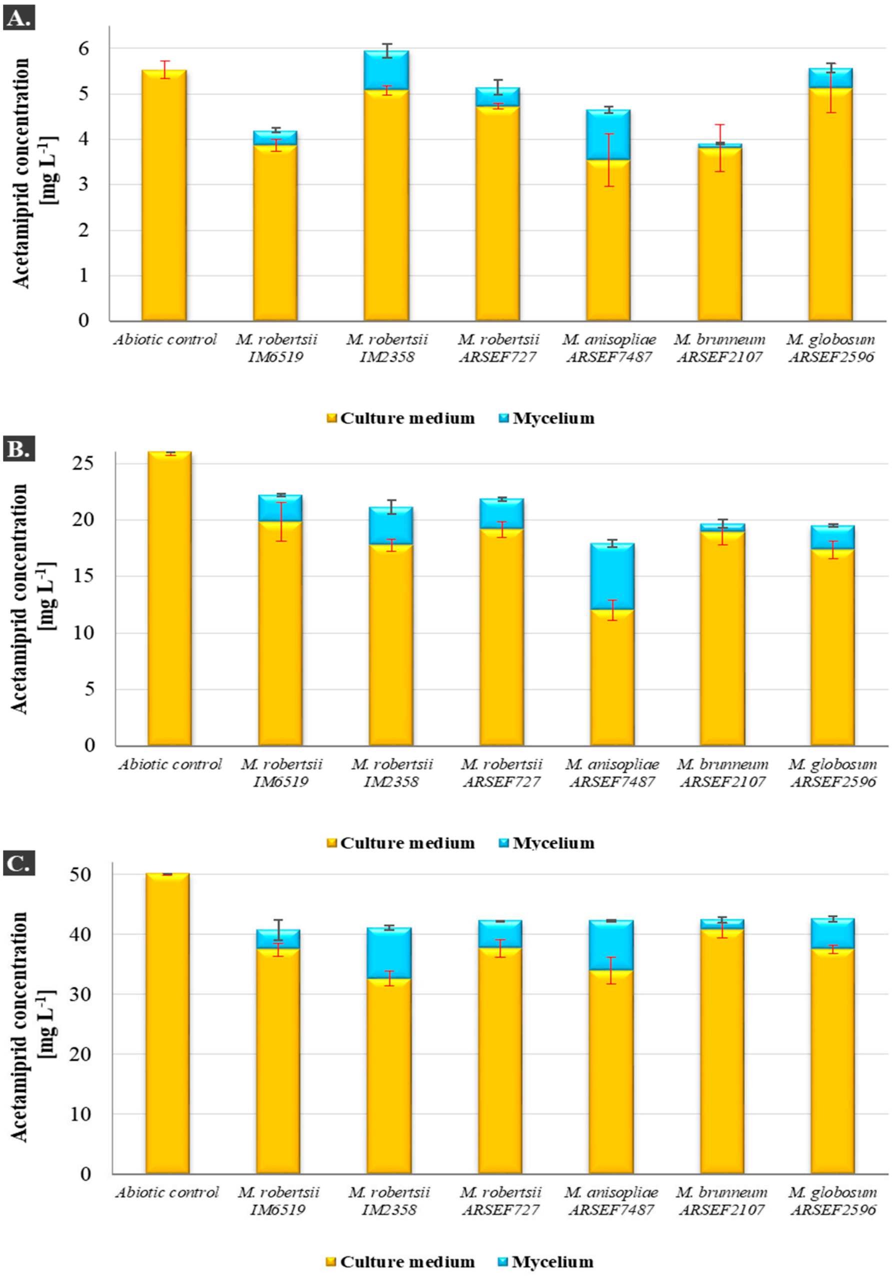

Figure S1. Elimination of acetamiprid at the concentrations of 5 **(A)**, 25 **(B)** and 50 mg L^{-1} **(C)** after 7 days of incubation by *Metarhizium* sp. considering the residual content in the culture medium and mycelium.

Table S1. Effect of acetamiprid on the permeability of the Metarhizium brunneum ARSEF2107 fungal cell wall.

	**Propidium Iodide Fluorescence**
**Control**	3162.01 ± 306.53
ACET 5 mg L^{-1}	3755.66 ± 527.62
ACET 25 mg L^{-1}	3877.92 ± 559.02
ACET 50 mg L^{-1}	3643.73 ± 318.41

Table S2. Multiple Reaction Monitoring (MRM) parameters for determining 19 types of destruxins.

**No.**	**Destruxin**	**Q1 Mass (Da)**	**Q3 Mass (Da)**	**Retention Time (min)**	**Collision Energy [V]**	**Declustering Potential [V]**	**Entrance Potential [V]**	**Collision Cell Exit Potential [V]**
**1.**	A	578	437<br>178	3.19	41<br>51	106	10	14
**2.**	B	594	453<br>194	3.28	45<br>38	106	10	14
**3.**	C	610	469<br>210	2.89	40<br>32	106	10	14
**4.**	D	624	483<br>224	2.97	30<br>40	106	10	14
**5.**	Ed	612	417<br>212	3.04	32<br>40	106	10	14
**6.**	F	596	455<br>196	3.1	38<br>45	106	10	14
**7.**	A1	592	451<br>192	3.24	38<br>45	106	10	14
**8.**	B1	608	467<br>208	3.15	32<br>40	106	10	14
**9.**	Ed1	626	485<br>226	2.96	30<br>40	106	10	14
**10.**	A2	564	423<br>178	3.13	43<br>51	106	10	14
**11.**	B2	580	439<br>194	3.23	41<br>45	106	10	14
**12.**	D2	610	469<br>224	2.88	32<br>40	106	10	14
**13.**	A3	566	425<br>166	3.18	43<br>53	106	10	14
**14.**	DesmA	564	437<br>178	3.12	41<br>51	106	10	14
**15.**	DesmB	580	453<br>194	3.24	38<br>45	106	10	14
**16.**	DesmC	596	469<br>210	3.08	32<br>40	106	10	14
**17.**	DesmB2	566	439	3.2	41	106	10	14

			194		45			
**18.**	CL	630	489<br>230	3.12	30<br>40	106	10	14
**19.**	E2CL2	616	475<br>230	3.07	32<br>40	106	10	14
